# Prevalence and Risk Factors of Infertility at a Rural Site of Northern China

**DOI:** 10.1371/journal.pone.0155563

**Published:** 2016-05-13

**Authors:** Jimei Cong, Pingping Li, Liqiang Zheng, Jichun Tan

**Affiliations:** 1 Assisted Reproduction Center, Obstetrics and Gynecology Department, Shengjing Hospital affiliated to China Medical University, Shenyang, Liaoning, China; 2 Epidemiology Department, Shengjing Hospital affiliated to China Medical University, Shenyang, Liaoning, China; University of Sydney, AUSTRALIA

## Abstract

**Objective:**

To investigate and analyze prevalence and risk factors of infertility at a representative rural site of Northern China.

**Method:**

This is a cross-sectional study. We conducted a face-to-face questionnaire survey from July 2014 to October 2014 involving 5,131 women who were at childbearing age in Suizhong, a medium-sized, representative county located in Northern China. Finally, data from 4,232 valid questionnaires were analyzed.

**Definition:**

Infertility is defined as the failure to achieve a clinical pregnancy after 12 months or more of regularly unprotected sexual intercourse.

**Results:**

Infertility prevalence in Suizhong County was 13.09% (95% CI, 12.09%-14.1%), of which the primary infertility incidence was 0.99% (95% CI, 0.72%-1.34%), and the secondary infertility incidence was 12.10% (95% CI, 11.13%-13.12%). For women, the infertility incidence of underweight women (Body Mass Index, BMI<18.5 kg/m^2^) was 1.5-fold higher than that of women with moderate BMI (18.5–24.9 kg/m^2^). The infertility incidence of women with little exercise was 4 times more than that of women with regular exercise, and 2 times more than that of women with heavy exercise. The group with moderate menstrual flow had the lowest prevalence of infertility, while both scant and excessive menstruation led to increased infertility incidence. Number of pregnancies (OR = 0.63; 95% CI, 0.51–0.79) was a protective factor for infertility, while the number of abortions (OR = 2.15; 95% CI, 1.58–2.93) was a risk factor for infertility. For men, those who stayed up late at night more than 3 times per week showed a significantly higher infertility incidence. Men who engaged in occupations with high-temperature working environment also suffered from an infertility incidence of about four times more than the others.

**Conclusions:**

We found significant association between women's infertility incidence with their BMI, state of exercise, amount of menstrual flow, number of pregnancies and number of abortions. As for men, both staying up late and engaging in high-temperature occupations are independent factors affecting their fertility.

## Introduction

Infertility is a pivotal issue for couples of childbearing age all over the world. Due to lifestyle changes and the presence of various environmental stress, the incidence of infertility increased significantly and has become the third most serious disease, following cancer and cardiovascular diseases. Infertility is a special reproductive health defect that is different from other diseases. It is not life threatening, but the detrimental influence of infertility to patients, their families and the whole society should not be underestimated [1–2].

It has been reported that the infertile couples worldwide account for 10% to 15% of all married couples [3]. A systematic analysis of infertility incidence in more than 190 countries and regions around the world showed that in 2010, women at the age of 20–44 years suffered from a primary infertility incidence of 1.9%, and a secondary infertility incidence of 10.5% [4]. Because of the huge impact of infertility on human reproductive health, such risk factors have attracted much concern. However, comprehensive epidemiological studies on the risk factors for infertility are not well documented [5–6]. Conventionally, parameters such as age, obstetrical history, smoking and drinking patterns, menstruation, BMI index, lifestyle and environmental factors are considered to be the major risk factors leading to infertility [7]. However, it is still worth finding out the specific risk factors of infertility in certain defined regions. In this study, we investigated and analyzed the prevalence of infertility and its risk factors in rural areas of northern China in order to provide guidance for the prevention and treatment of infertility.

## Materials and Methods

This study was approved by the ethics committee of Shengjing Hospital affiliated to China Medical University. All participants signed the consent forms. The original data was stored in the database of Shengjing Hospital affiliated to China Medical University. All data were analyzed anonymously.

### Study population

**Fig 1:**
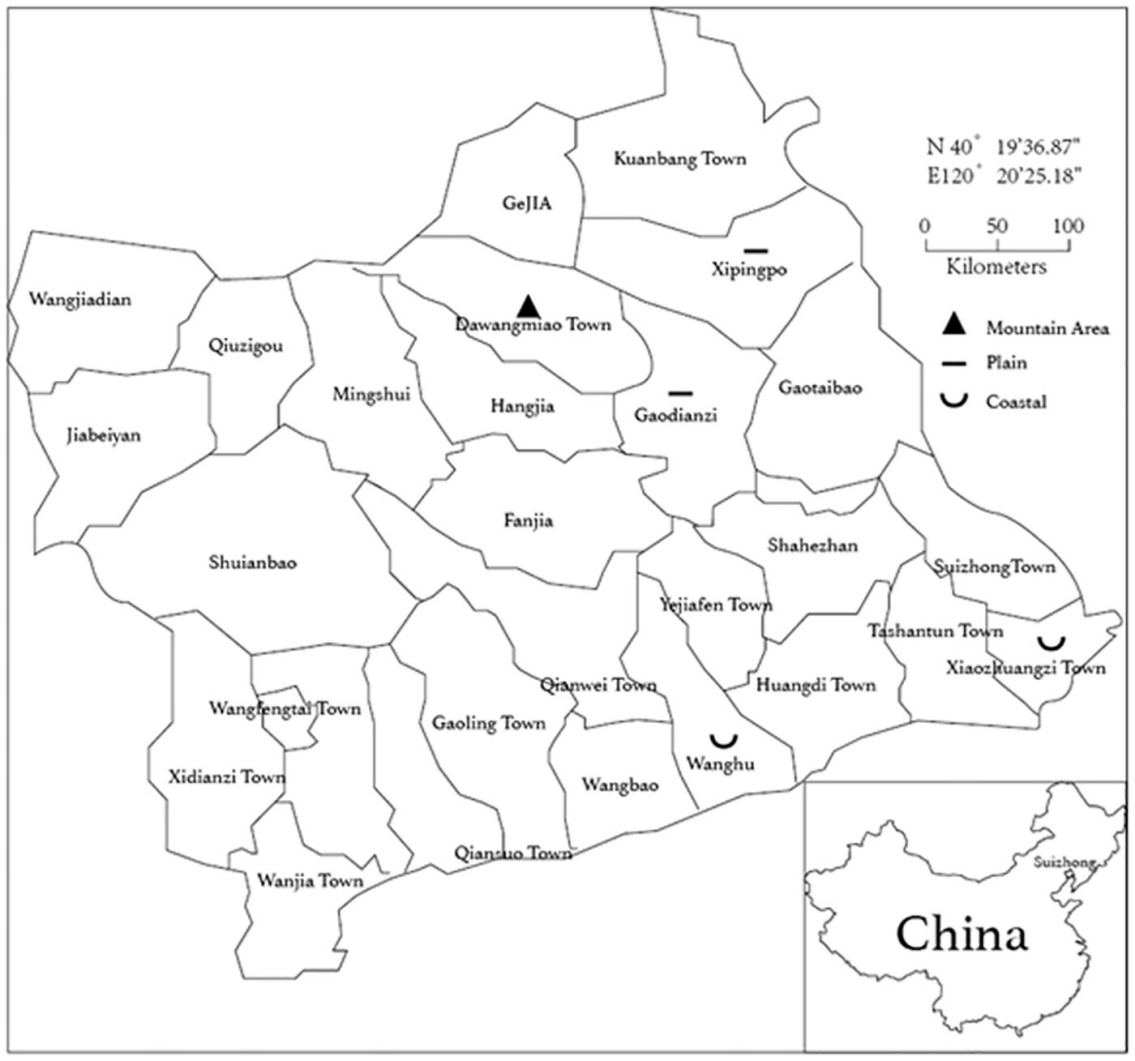
The five selected towns in Suizhong County. We stratified the entire 25 areas of Suizhong County in three layers in accordance with the coast, plains, and the mountainous area; and then we randomly chose five towns (two from coastal regions, one from the plains, and one from the mountainous area).

This is a cross-sectional study. The inclusion criteria are as follows: (1) the date of birth of the participants was confined to between July 1964 and July 1994 (aged 20–49 years); (2) the female participants should be in a *de-facto* marriage; and (3) the participants should be local resident. The exclusion criteria are as follows: (1) the participant was divorced or widowed; and (2) the couple was separated>6 months/year. The survey was conducted from July 2014 to October 2014. Suizhong County (Fig 1), the selected site for this survey, has a population of 670,000. It has a long and narrow terrain and a diverse topography, representing typical rural areas of northern China. Stratified cluster sampling was used in this study and we stratified the entire 25 areas of Suizhong County in three layers in accordance with the coast, plains, and the mountainous area. Then we randomly chose five towns (two from coastal regions, one from the plains, and one from the mountainous area). In the five selected towns (total population about 150,000), we further randomly selected four to six villages as our final sampling sites from each area according to their populations. A total of 22 villages from Suizhong County were investigated in this survey. This study was approved by the regional ethical committee.

### Data collection

In the present study, we employed a self-designed questionnaire to collect the following information: (1) general demographic information of both male and female participants, including age, address, education, occupation, height, weight, etc. BMI was calculated as weight in kilograms divided by the square of the height in metres (kg/m^2^) according to the WHO criteria, which defined underweight as less than 18.5 kg/m^2^; moderate as 18.5–24.9 kg/m^2^; overweight as 25.0–29.9 kg/m^2^; and obese as greater than 30 kg/m^2^ [8]; (2) marriage and childbearing status, including the age at marriage, marriageable age, number of pregnancies and abortions, etc.; (3) disease history; (4) menstruation status, including whether it is regular or not, the presence or absence of dysmenorrhea, and the amount. According to the Williams Gynecology definition, normal menstrual blood volume is 30 to 50 ml, while 20 ml or less is considered as scanty and more than 80 ml is defined as excessive menstruation; and (5) information on their living habits including exercise amounts, etc. We used a classification scheme according to the amount of exercise, with light exercise defined as less than 3 times/week, and moderate exercise as more than 1h and at least 3 times per week. The women who participated in intense athletic competition or were engaged in athletics-related occupations were classified as performing heavy exercise [9–10]. The data were analyzed anonymously.

### Quality control

We achieved a strict quality control by performing a pilot-survey ahead of the experiment, the investigator-unified training, supervision and audition of the questionnaire were processed throughout the entire study. To ensure the quality of our final survey, we conducted a pilot survey in July 2014 in randomly selected 22 villages. We held several meetings to discuss our findings, to add or delete items and to adjust the structure of the questionnaire according to the results from the pilot survey, and eventually generated a final questionnaire. A total of 36 investigators participated in this household survey, who are doctors and staff from the local Health and Epidemic Prevention Station with medical education background and are familiar with the residents' marriage status and their childbearing status. During the investigation, we also received support from the Suizhong County Health Department. Steering groups at all levels assisted in the dissemination and mobilization work, and supervised the implementation of our survey. We randomly sampled 5% of the households surveyed to validate their authenticity.

### Definition of infertility

Although there are numerous epidemiological studies related to infertility, the definition of infertility remains controversial. Recent reports on the incidence of infertility delineate the following definitions: 1) current infertility was defined as couples who were currently infertile if they did not become pregnant after exposure to the risk of conception during the previous 12 months; 2) subfecundity was defined as prolonged time-to-pregnancy and difficulty in carrying the pregnancy to a live birth; 3) childless was defined as not giving birth within a certain period after marriage; and 4) lifetime infertility/cumulative infertility was defined as ever having had difficulty in conceiving [10]. Current infertility is generally less prevalent than lifetime infertility, as the latter represents a lifelong feature [11]. The definition of current infertility was employed in this survey study.

We used the WHO definition of infertility. Infertility is defined as the failure to achieve a clinical pregnancy after 12 months or more of regularly unprotected sexual intercourse [12]. Female primary infertility: women who have never been pregnant or given birth after sexual maturity. Female secondary infertility: women who are having difficulty in becoming pregnant with previous successful delivery.

Prevalence refers to the percentage of infertility among the entire population at a particular time point.

### Statistical analysis

After data collection, we chose to analyze the questions of response rate over 80%. Some questions with less than 80% response rate were abandoned due to the understanding ability or privacy of people asked.

The statistical analysis of the absolute number of infertile occurrences and the incidence of infertility was conducted using the Epidata 3.0 database with double dual entry. Quantitative data are presented as mean ± standard deviation. When variance between groups showed homogeneity, we compared different groups using the t-test; otherwise, we used the t'-test. Rating data between groups were evaluated by comparing the median (inter-quartile range) with Wilcoxon's test. Qualitative data were analyzed by frequency (percentage), and groups were compared using the χ^2^ test.

To analyze the absolute number and the incidence of infertility, we calculate the 95% confidence interval based on the binomial distribution. The incidence among populations was compared using the χ^2^ test.

We also developed a univariate logistic regression model between the prevalence of infertility and the factors studied. To verify the statistically significant indicators, we used the method of stepwise back forward to generate a multivariate logistic regression model. The stepwise regression inclusion criterion was p≤0.05, and the exclusion criterion was p>0.05. Statistical analysis was performed using SAS 9.2 software (SAS Institute Inc., Cary, NC, USA). Figures were created using STATA 13.0 software (StataCorp, College Station, Texas, USA).

## Results

### General demographic characteristics

As shown in Table 1, the mean age was 37.50 years for the female infertility group, and 38.48 years for the non-infertility group. On the other hand, the mean age was 38.94 years for the male infertility group, and 39.78 years for the non-infertility group. The difference of the mean age between men and women was statistically significant (P = 0.01). Regarding the geographic characteristics of the participants sampled, 156 (28.16%) infertile couples came from the plains, 128 (23.1%) came from the mountainous area, and 270 (48.74%) came from the coastal area, and such variations are statistically significant (P<0.0001). Compared with the non-infertility group, the infertility counterpart exhibited an older age at marriage, a longer marriageable age, fewer pregnancies, and more abortions (Table 1). Women's weight, height and BMI between the two groups were not statistically different.

**Table 1:** General demographic characteristics of Suizhong County.

Factors	Non-infertility	Infertility	P value
**Total number**	3678	554	
**Female age**	38.48 ± 7.77	37.50 ± 8.18	0.0058
**Male age**	39.78 ± 7.90	38.94 ± 8.53	0.0290
**Terrain**			
*Plains*	1057 (28.74%)	156 (28.16%)	<.0001
*Mountains*	1192 (32.41%)	128 (23.10%)	
*Coastal*	1429 (38.85%)	270 (48.74%)	
**Age at marriage**	21.93 ± 2.38	22.30 ± 2.46	0.0008
**Marriage age limit**	17.00 ± 7.61	15.49 ± 8.06	<.0001
**Number of pregnancies**	1.95 ± 0.85	1.84 ± 0.90	0.0077
**Number of abortions**	0.26 ± 0.57	0.37 ± 0.69	0.0001
**Weight of women**	60.71 ± 7.32	60.98 ± 8.89	0.4481
**Height of women**	160.89 ± 4.42	160.85 ± 4.63	0.8378
**Female BMI (kg/m**^**2**^**)**	23.45 ± 2.69	23.56 ± 3.25	0.3964

Abbreviations: BMI, body mass index, calculated as weight in kilograms divided by height in meters squared. Values are given as mean ± SD or percentage.

### Prevalence of infertility

A total of 129,422 residents were questioned in this survey. The 22 villages in our final sampling sites hosted 5,131 women of childbearing age, based on the data of the local family planning departments. We ultimately received a total of 4,466 questionnaires, of which 4,232 were valid. The response rate was 87%, with a pass rate of 94.8%. There were 554 cases of infertility, with an infertility incidence of 13.09% (95% CI, 12.09%-14.14%). Among which, 42 cases were of primary infertility, with an incidence of 0.99% (95% CI, 0.72%-1.34%), while 512 cases of secondary infertility, with an incidence of 12.10% (95% CI, 11.13%-13.12%).

### Various study factors and infertility prevalence

**Fig 2:**
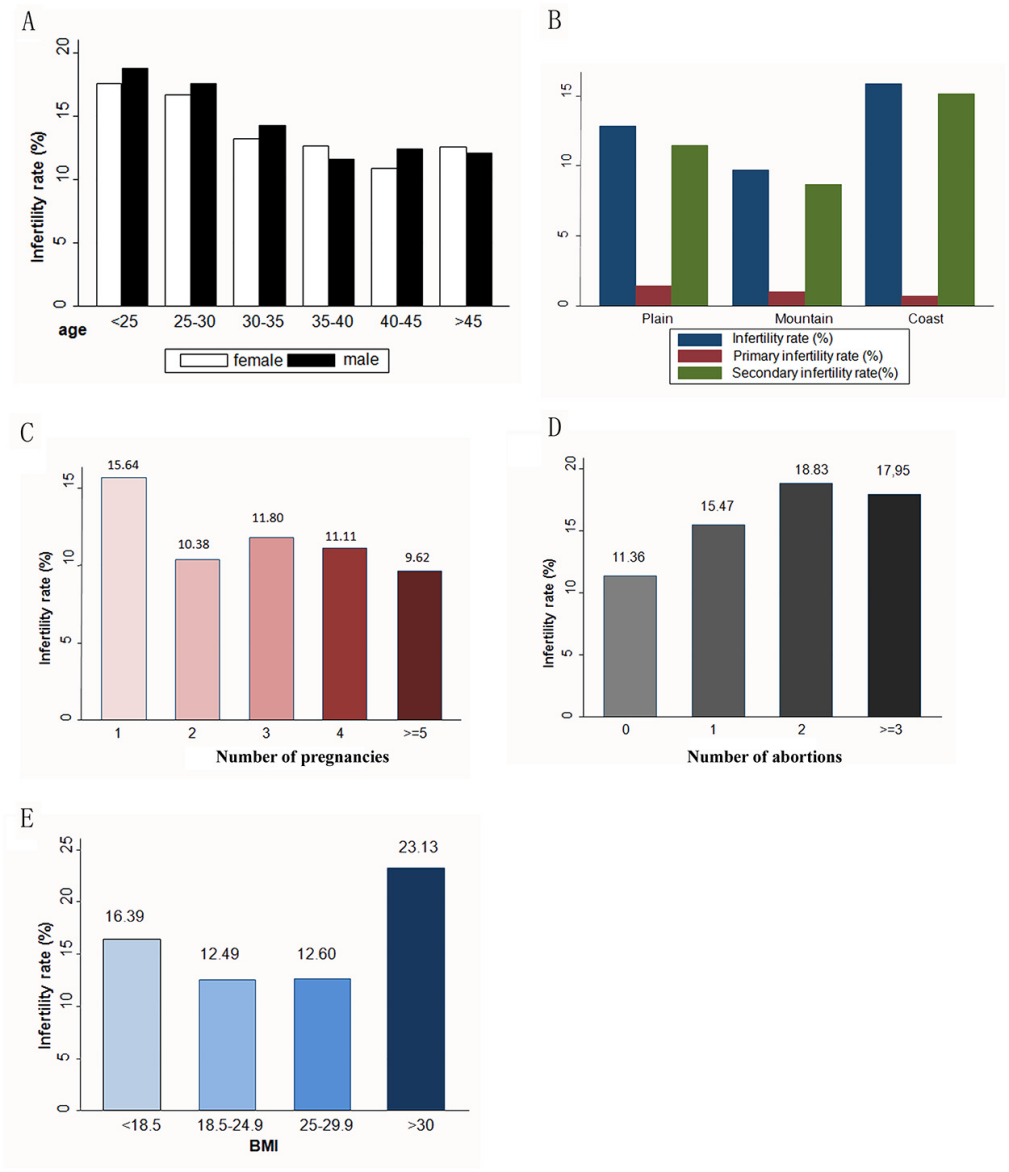
**A) Age and infertility incidence:** Male and female infertility incidences for all age groups are shown in this figure. Overall, both male and female age contribute to the incidence of infertility positively. **B) Terrain and infertility incidence:** Infertility incidences among couples living in different terrains vary significantly, especially for the secondary infertility incidence. The highest incidence of infertility occurred in those who live in coastal areas, followed by those who live plains and mountainous areas. **C) Number of pregnancies and infertility incidence:** With the increase in the number of pregnancies, we observed a concomitant drop of the infertility incidence. **D) Number of abortions and infertility incidence:** The increase in the number of abortions correlated with the incidence of infertility positively. **E) BMI and infertility incidence:** Incidence of infertility is lower in relatively moderate women BMI group, women with both underweight and obese will suffer high infertility incidence.

Male and female infertility incidence for all age groups were shown in Fig 2A. We further analyzed the infertility incidence of different age groups and decided to place all age groups into the model as grade variables. We found that with the increase of the female age at one level, the risk of infertility changed to 0.92 times of the original value (95% CI, 0.87–0.97; P = 0.0028). With the increase of the male age at one level, the risk of infertility changed to 0.91 times of the original value (95% CI, 0.85–0.96; P = 0.0015). Thus, both male and female age contribute to the incidence of infertility positively. At the same time, we compared the infertility incidence among couples living in different terrains and found that the highest incidence of infertility occurred in those who live in coastal areas, followed by those who live in plains and mountainous areas (Fig 2B). Interestingly, the primary infertility incidence in all these three areas were quite similar, whereas the secondary infertility incidence in mountains, plains and coast were significantly different.

The relationship between the number of pregnancies/abortions of the local female residents at the child-bearing age and their infertility incidence is shown in Fig 2C and 2D.

With the increase in the number of pregnancies, we observed a concomitant drop of the infertility incidence. The increase in the number of abortions correlated with the incidence of infertility positively. The P values for the trend analysis test with regard to each index were <0.05.

The relationship between BMI values of women at childbearing age and the incidence of infertility was as follows (Fig 2E): underweight women (BMI less than 18.5 kg/m^2^) had an infertility incidence of 16.39%; the infertility incidence in moderate women (BMI 18.5–24.9 kg/m^2^) was lowest (12.49%); and in overweight women (BMI 25–29.9 kg/m^2^) the infertility incidence was 12.60%. Obese women whose BMI were greater than 30 kg/m^2^, had an infertility incidence of up to 23.13%. It can be concluded that women with moderate BMI had the lowest incidence of infertility, and the overweight group was second. Underweight and obese women had high incidences of infertility, and the incidence of infertility was highest in the obesity group.

### Risk factors of infertility

After adjustment for other relevant factors, we generated a multivariate logistic regression model to analyze the independent risk factors for infertility. We listed the risk factors with a strong impact on infertility incidence in Table 2, among which BMI is a significant factor affecting the incidence of infertility. Compared to the moderate BMI group (18.5–24.9 kg/m^2^), underweight women (BMI<18.5 kg/m^2^) were rendered a 1.5-fold increase in the infertility incidence. In addition, the infertility prevalence of obese women (BMI>30 kg/m^2^) was up to 2.3 times greater than those with moderate BMI.

**Table 2:** Risk factors for infertility.

Factor	OR	P value	95%CI
**Female BMI (kg/m**^**2**^**)**			
18.5–24.9	1.0 reference		
<18.5	1.51	0.1464	0.87, 2.64
25–29.9	0.82	0.1971	0.60, 1.11
>30	2.30	0.0041	1.30, 4.07
**Female exercise**			
Light	1.0 reference		
Regular	0.25	0.0043	0.10, 0.65
Heavy	0.58	0.0015	0.42, 0.81
**Menstruation flow**			
Moderate	1.0 reference		
Scanty	1.32	0.1437	0.91, 1.93
Excessive	2.02	0.0349	1.05, 3.87
**Male staying up late at night**			
<twice/week	1.0 reference		
2–3 times/week	0.60	0.0132	0.40, 0.90
>3 times/week	1.55	0.3717	0.59, 4.09
**Men engaged in high-temperature occupations**			
Yes	1.0 reference		
No	0.28	0.0047	0.11, 0.67
**Number of pregnancies**	0.63	<.0001	0.51, 0.79
**Number of abortions**	2.15	<.0001	1.58, 2.93

Abbreviations: OR, odds ratio; BMI, body mass index; CI, confidence interval. Adjust for all other variables in the table, age, terrain (plains, mountains, coastal), age at marriage, marriage age limit, weight and height of women.

Exercise amount was found to affect infertility too. Comparison among the three groups showed that the infertility incidence was lowest in regularly exercising women, while women who were lightly exercised showed 4 times more infertility incidence. The heavily exercising group followed, accounting for 2 times more than the prevalence of the regularly exercising group.

The other important risk factor is menstrual blood volume. Therefore we divided the sampled women into three groups: scanty (<20 ml), moderate (20–80 ml), and excessive (>80 ml). Our results showed that the group with moderate menstrual flow exhibited the lowest prevalence of infertility, and both scanty and excessive menstruation led to an increase in the infertility incidence.

It can be seen from Table 1 that men staying up late at night more than 3 times/week, showed an increased infertility incidence. Men who were engaged in high-temperature occupations showed an infertility incidence about four times more than that of others. Number of pregnancies (OR = 0.63; 95% CI, 0.51–0.79) was a protective factor from infertility, while the number of abortions (OR = 2.15; 95% CI, 1.58–2.93) was a risk factor for infertility.

Finally, by mutually analysis, we found that among people younger than 35 years old, exercise amount of women and number of abortions are the risk factors for infertility (Table A in S1 File), while BMI, women exercise amount, number of abortions, number of pregnancies, men staying up late at night and men engaging in high-temperature occupations are all risk factors for infertility in people older than 35 years old (Table B in S1 File). The mainly risk factor of primary infertility is BMI (Table C in S1 File), while BMI, women exercise amount, menstruation flow, number of abortions, number of pregnancies, men staying up late at night and men engaged in high-temperature occupations are all risk factors for secondary infertility (Table D in S1 File).

## Discussion

The incidence of infertility varies greatly in different countries and regions. A U.S. survey depicted 15,303 married women of 15–44 years old as having a 7.4% current infertility in 2002 [13]. In 2003, Tanzania surveyed 2019 women aged 20–44 years, and their current infertility incidence was 6.9% [14]. In a cross-sectional study conducted in 2004 entailing a population of more than 495,000 women, the five-year primary infertility incidence was 18.8%, and secondary infertility was 0.8%-21.6% [15]. The current infertility incidence was 14.2% in an Indian survey in 2007 [16]. Cumulative and current infertility incidences were 24.9% and 3.4%, respectively, in a study of 10,783 women in Iran in 2009 [17]. Canada surveyed 4,412 women in 2010, and described a 15.6% current infertility incidence [12]. The infertility incidence in 2,151 newly married couples in China was 14.2% in 2012 [5]. Presently, reports on incidence of infertility in rural areas in northern China are still quite rare. Our survey showed that the primary infertility incidence was 0.9% in rural areas in Northern China, significantly lower than most results reported for other regions in China. However, the secondary infertility prevalence was 12.1%, which was similar to other results reported previously [5, 15]. We assume that the low primary infertility may be due to the less environmental pollution, young childbearing age in rural areas, and less adverse environmental exposure. The secondary infertility incidence reported, although similar to most results reported for China, was relatively high compared with the primary infertility incidence. This may be explained by the fact that medical conditions continue to be lagged behind in these rural areas, self-care for women is relatively poor, and women are more prone to suffer from the secondary infertility. The higher abortion incidence in Suizhong County is also a primary reason for the high secondary infertility incidence. Most domestic and foreign reports have revealed a positive correlation between the prevalence of infertility and age [18]. However, in our study the infertility incidence for both men and women was reduced with increasing age. We postulated that people born later may be exposed to more harmful environments and may also develop poorer living habits. A woman's age at marriage was also a factor influencing infertility. The age at marriage in women from the infertile group was greater than that of the fertile group. Based on this finding, we advocate for age-appropriate marriages.

With overall improvements to the economy and changes to our lifestyles, the number of overweight and underweight individuals is increasing, and obesity is an important factor leading to infertility. Esmaeilzadeh *et al* found in their study that infertile women had a 4.8-fold increased risk of obesity and almost a 3.8-fold increased risk of being overweight compared to fertile women [19]. Our survey showed that both underweight and obesity are risk factors for infertility. In the present study, primary and secondary infertility incidence were higher in women with a BMI of less than 18.5 kg/m2. We postulated that this may be related to gonadal function and a fertility decline caused by excessive weight loss, and anovulation caused by long-term inadequate nutritional intake. Infertility prevalence for obese women (BMI>30 kg/m^2^) was up to 2.3 times greater than for the moderate-BMI group, which may be related to abnormal metabolism caused by obesity. Obesity can cause ovarian dysfunction resulting in ovulatory disorder, eventually leading to reduced fertility incidence. However, studies have shown that infertility caused by ovulation disorders in obese women was reversible, and 90% of previously anovulatory obese women began ovulating after losing an average weight of 10.2 kg [20]. Moreover, studies also found that with increasing BMI, embryonic implantation incidence was also reduced. Compared with non-obese women, the ongoing pregnancy incidence per cycle was only 38.3% in obese women, while in the former group it was up to 45.5% [21]. At the same time, we additionally found that appropriate exercise reduced the incidence of infertility. Therefore, proper exercise and the maintenance of a moderate weight are the key factors to reduce infertility prevalence.

Our study showed that high-temperature occupations should also be listed as risk factor for male infertility. Sperm density, mobility and number of morphologically normal sperms were significantly lower in a high-temperature environment [22]. Although, the decline in sperm quality due to high temperatures was reversible [23]. We suggest that men who wish to father a child avoid exposure to high temperatures.

The number of pregnancies is also a protective factor from infertility, and the number of abortions is a risk factor for infertility. Intrauterine environmental considerations such as recurrent miscarriage and placement and removal of IUDs (intrauterine devices) are correlated with reproductive system infections, especially pelvic infections. An early study confirmed that pelvic infection is a vital factor leading to female infertility [24]. Female pelvic infection can cause pelvic inflammatory disease and thus the occurrence of pelvic adhesions, resulting in infertility [25], and negative-pressure operations during abortions may also cause immune infertility. Therefore, in order to reduce the incidence of infertility, we should also expand our knowledge on contraception so as to avoid the occurrence of unwanted pregnancy, and pay greater attention to the dangers of abortion and to advocate overall safe medical treatment.

Collectively, though risk factors for infertility are multiple and complex, and the investigated place couldn't represent all the situations in china rural regions, we expect that the results from the present study will contribute to the improvement of the overall reproductive health of habitants in China rural areas. We will continue spare no effort to provide a theoretical basis for the prevention and treatment of infertility.

## Supporting Information

S1 File **Table A in S1 File:** The association results between overall infertility and each risk factor derived from logistic regression model in samples with age < 35 years. **Table B in S1 File:** The association results between overall infertility and each risk factor derived from logistic regression model in samples with age >35 years. **Table C in S1 File:** The association results between primary infertility and each risk factor derived from logistic regression model in overall samples. **Table D in S1 File:** The association results between secondary infertility and each risk factor derived from logistic regression model in overall samples.
(DOC)
