# Supplementary material for: Prevalence and Risk Factors of Infertility at a Rural Site of Northern China
Source: PLoS One. 2016 May 13;11(5):e0155563. doi: 10.1371/journal.pone.0155563 (PMC4866679; doi:10.1371/journal.pone.0155563)
Supplement: S1 File — Table A in S1 File: The association results between overall infertility and each risk factor derived from logistic regression model in samples with age < 35 years. Table B in S1 File: The association results between overall infertility and each risk factor derived from logistic regression model in samples with age >35 years. Table C in S1 File: The association results between primary infertility and each risk factor derived from logistic regression model in overall samples. Table D in S1 File: The association results between secondary infertility and each risk factor derived from logistic regression model in overall samples. (DOC) [file pone.0155563.s001.doc]

| Table A in S1 File: The association results between overall infertility and each risk factor derived from logistic regression model in samples with age < 35 years | | | | | | |
| --- | --- | --- | --- | --- | --- | --- |
| Factor | Univariate logistic regression | | | Multivariate logistic regression | | |
|  | OR* | 95％CI | P* | OR* | 95％CI | P* |
| Female BMI |  |  |  |  |  |  |
| 18.5-24.9 | reference |  |  | reference |  |  |
| <18.5 | 1.6536 | 0.9286,2.9445 | 0.0875 | 1.6122 | 0.7011,3.7076 | 0.2610 |
| 25-29.9 | 1.3514 | 0.9409,1.9410 | 0.1031 | 0.7916 | 0.4210,1.4885 | 0.4683 |
| >30 | 3.0594 | 1.5906,5.8845 | 0.0008 | 2.4600 | 0.9032,6.6998 | 0.0783 |
| Female exercise |  |  |  |  |  |  |
| Light | reference |  |  | reference |  |  |
| Regular | 0.9054 | 0.3052,2.6854 | 0.8578 | 1.2838 | 0.3298,4.9970 | 0.7187 |
| Heavy | 0.6973 | 0.4605,1.0558 | 0.0885 | 0.4305 | 0.2228,0.8320 | 0.0122 |
| Menstruation flow |  |  |  |  |  |  |
| Moderate | reference |  |  | reference |  |  |
| Scanty | 1.3178 | 0.8018,2.1660 | 0.2764 | 1.8615 | 0.9054,3.8273 | 0.0911 |
| Excessive | 1.2112 | 0.4917,2.9834 | 0.6770 | 2.1487 | 0.6427,7.1840 | 0.2142 |
| Male staying up late at night |  |  |  |  |  |  |
| <twice/week | reference |  |  | reference |  |  |
| 2-3 times/week | 0.5974 | 0.3569,1.0001 | 0.0500 | 0.7117 | 0.3261,1.5533 | 0.3931 |
| >3times/week | 1.7188 | 0.5448,5.4234 | 0.3555 | 1.9517 | 0.3900,9.7662 | 0.4157 |
| Men engaged in high-temperature occupations | | |  |  |  |  |
| Yes | reference |  |  | reference |  |  |
| No | 0.4438 | 0.1543,1.2759 | 0.1316 | 0.3639 | 0.0559,2.3695 | 0.2903 |
| Number of pregnancies | 0.8819 | 0.6847,1.1360 | 0.3307 | 0.6393 | 0.3702,1.1042 | 0.1086 |
| Number of abortions | 1.3750 | 1.0115,1.8690 | 0.0421 | 2.1478 | 1.0530,4.3810 | 0.0356 |

| Table B in S1 File: The association results between overall infertility and each risk factor derived from logistic regression model in samples with age >35 years | | | | | | |
| --- | --- | --- | --- | --- | --- | --- |
| Factor | Univariate logistic regression | | | Multivariate logistic regression | | |
|  | OR* | 95％CI | P* | OR* | 95％CI | P* |
| Female BMI |  |  |  |  |  |  |
| 18.5-24.9 | reference |  |  | reference |  |  |
| <18.5 | 1.0814 | 0.5930,1.9720 | 0.7986 | 1.4296 | 0.6572,3.1099 | 0.3675 |
| 25-29.9 | 0.9281 | 0.7350,1.1718 | 0.5305 | 0.8193 | 0.5720,1.1736 | 0.2771 |
| >30 | 1.7504 | 1.0745,2.8517 | 0.0246 | 2.2826 | 1.1273,4.6222 | 0.0219 |
| Female exercise |  |  |  |  |  |  |
| Light | reference |  |  | reference |  |  |
| Regular | 0.1664 | 0.0525,0.5279 | 0.0023 | 0.1124 | 0.0263,0.4807 | 0.0032 |
| Heavy | 1.0064 | 0.7616,1.3298 | 0.9644 | 0.6373 | 0.4286,0.9475 | 0.0260 |
| Menstruation flow |  |  |  |  |  |  |
| Moderate | reference |  |  | reference |  |  |
| Scanty | 1.6430 | 1.2123,2.2267 | 0.0014 | 1.1690 | 0.7433,1.8383 | 0.4991 |
| Excessive | 1.9189 | 1.1739,3.1370 | 0.0093 | 1.9552 | 0.8911,4.2900 | 0.0945 |
| Male staying up late at night |  |  |  |  |  |  |
| <twice/week | reference |  |  | reference |  |  |
| 2-3 times/week | 0.9499 | 0.7011,1.2869 | 0.7400 | 0.5686 | 0.3486,0.9273 | 0.0237 |
| >3times/week | 3.0880 | 1.4114,6.7562 | 0.0048 | 1.4124 | 0.3839,5.1965 | 0.6034 |
| Men engaged in high-temperature occupations | | |  |  |  |  |
| Yes | reference |  |  | reference |  |  |
| No | 0.5166 | 0.2463,1.0839 | 0.0806 | 0.2571 | 0.0916,0.7212 | 0.0099 |
| Number of pregnancies | 0.8415 | 0.7285,0.9721 | 0.0190 | 0.6443 | 0.4880,0.8506 | 0.0019 |
| Number of abortions | 1.3286 | 1.1279,1.5651 | 0.0007 | 2.1132 | 1.4752,3.0271 | <.0001 |

| Table C in S1 File: The association results between primary infertility and each risk factor derived from logistic regression model in overall samples | | | | | | |
| --- | --- | --- | --- | --- | --- | --- |
| Factor | Univariate logistic regression | | | Multivariate logistic regression | | |
|  | OR* | 95％CI | P* | OR* | 95％CI | P* |
| Female BMI |  |  |  |  |  |  |
| 18.5-24.9 | reference |  |  | reference |  |  |
| <18.5 | 2.5147 | 0.7248,8.7248 | 0.1463 | 3.3879 | 0.9018,12.7275 | 0.0708 |
| 25-29.9 | 1.4674 | 0.7230,2.9781 | 0.2883 | 1.5126 | 0.6321,3.6194 | 0.3526 |
| >30 | 6.2575 | 2.4064,16.2721 | 0.0002 | 4.3293 | 1.1439,16.3847 | 0.0309 |
| Female exercise |  |  |  |  |  |  |
| Light | reference |  |  | reference |  |  |
| Regular | 1.3737 | 0.3116,6.0561 | 0.6749 | 1.3341 | 0.2497,7.1276 | 0.7360 |
| Heavy | 1.6027 | 0.7595,3.3819 | 0.2157 | 1.4900 | 0.5910,3.7560 | 0.3980 |
| Menstruation flow |  |  |  |  |  |  |
| Moderate | reference |  |  | reference |  |  |
| Scanty | 1.9925 | 0.8646,4.5919 | 0.1056 | 2.3534 | 0.8674,6.3851 | 0.0928 |
| Excessive | 1.9041 | 0.4482,8.0883 | 0.3829 | 2.5445 | 0.5668,11.4230 | 0.2229 |

The 4 variables (Male staying up late at night, Men engaged in high-temperature occupations, Number of pregnancies and Number of abortions) did not be included in the model due to none or few primary infertility cases in the subgroups.

| Table D in S1 File: The association results between secondary infertility and each risk factor derived from logistic regression model in overall samples | | | | | | |
| --- | --- | --- | --- | --- | --- | --- |
| Factor | Univariate logistic regression | | | Multivariate logistic regression | | |
|  | OR* | 95％CI | P* | OR* | 95％CI | P* |
| Female BMI |  |  |  |  |  |  |
| 18.5-24.9 | reference |  |  | reference |  |  |
| <18.5 | 1.3073 | 0.8522,2.0055 | 0.2197 | 1.5124 | 0.8654,2.6433 | 0.1464 |
| 25-29.9 | 0.9832 | 0.8035,1.2031 | 0.8694 | 0.8165 | 0.6001,1.1111 | 0.1971 |
| >30 | 1.8671 | 1.2349,2.8229 | 0.0031 | 2.3017 | 1.3029,4.0661 | 0.0041 |
| Female exercise |  |  |  |  |  |  |
| Light | reference |  |  | reference |  |  |
| Regular | 0.2165 | 0.0881,0.5319 | 0.0009 | 0.2505 | 0.0969,0.6471 | 0.0043 |
| Heavy | 0.8391 | 0.6598,1.0672 | 0.1527 | 0.5827 | 0.4174,0.8133 | 0.0015 |
| Menstruation flow |  |  |  |  |  |  |
| Moderate | reference |  |  | reference |  |  |
| Scanty | 1.4868 | 1.1370,1.9441 | 0.0037 | 1.3244 | 0.9088,1.9300 | 0.1437 |
| Excessive | 1.6577 | 1.0628,2.5854 | 0.0258 | 2.0171 | 1.0509,3.8718 | 0.0349 |
| Male staying up late at night |  |  |  |  |  |  |
| <twice/week | reference |  |  | reference |  |  |
| 2-3 times/week | 0.8817 | 0.6752,1.1513 | 0.3550 | 0.5966 | 0.3966,0.8974 | 0.0132 |
| >3times/week | 2.8565 | 1.4949,5.4582 | 0.0015 | 1.5547 | 0.5904,4.0942 | 0.3717 |
| Men engaged in high-temperature occupations | | |  |  |  |  |
| Yes | reference |  |  | reference |  |  |
| No | 0.4911 | 0.2638,0.9142 | 0.0249 | 0.2773 | 0.1141,0.6741 | 0.0047 |
| Number of pregnancies | 0.8307 | 0.7372,0.9361 | 0.0023 | 0.6330 | 0.5053,0.7931 | <.0001 |
| Number of abortions | 1.3250 | 1.1474,1.5301 | 0.0001 | 2.1517 | 1.5778,2.9343 | <.0001 |
